# Multiple Endocrine Neoplasia Type 1 Presenting as Recurrent Overt Gastrointestinal Bleeding and Ulceration: A Diagnostic Challenge

**DOI:** 10.7759/cureus.99252

**Published:** 2025-12-15

**Authors:** Vetha Irene Sanjana, Jawairia Fahim, Asif Sekander, Rajaratnam Rameshshanker

**Affiliations:** 1 Internal Medicine, The Hillingdon Hospital, London, GBR; 2 Acute Medicine, The Hillingdon Hospital, London, GBR; 3 Gastroenterology, The Hillingdon Hospital, London, GBR

**Keywords:** endocrine, endocrine disorders, gi bleed, hypercalcemia, men 1, multiple endocrine neoplasia type 1 (men-1), neuroendocrine tumor, parathyroid adenoma

## Abstract

Multiple endocrine neoplasia type 1 (MEN1) is a multisystem endocrine disorder marked by tumors of the parathyroids, pancreatic islet cells, and pituitary gland. Although most cases are inherited through an autosomal dominant pattern, a proportion arises through sporadic mutation. As the clinical presentation is highly variable, it often leads to diagnostic delay in patients with de novo mutations. We report a 48-year-old man with no family history and a past medical history of renal calculi with intermittent hematuria. Over several months, he had multiple hospitalizations due to progressive epigastric pain and melena. Initial imaging suggested a localized small bowel perforation, which was treated conservatively. Endoscopic evaluation revealed severe esophagitis and duodenitis with biopsies suggesting peptic duodenitis. His symptoms were initially attributed to peptic ulcer disease and later to possible inflammatory bowel disease (IBD), and he was managed with proton pump inhibitors (PPIs) and steroids. Despite this, he continued to represent similarly and also developed an episode of upper-limb thrombophlebitis requiring anticoagulation. Biochemical investigations demonstrated persistent hypercalcemia, elevated parathyroid hormone, and significantly raised fasting gastrin levels whilst off high-dose PPI, raising suspicion for MEN1 syndrome. Functional imaging identified a parathyroid adenoma and multiple pancreatic and duodenal neuroendocrine tumors. Genetic testing confirmed a pathogenic MEN1 mutation. He was referred for parathyroidectomy followed by total pancreatectomy with duodenectomy, while first-degree relatives were offered genetic screening. This case highlights the diagnostic complexity of MEN1 in the absence of a family history, in which gastrointestinal ulcerations with bleeding, fleeting thrombophlebitis, and hypercalcemia may serve as early clinical clues.

## Introduction

Multiple endocrine neoplasia type 1 (MEN1) is caused by inactivating mutations of the tumor suppressor gene MEN1, which encodes the protein menin [1]. Loss of menin function results in abnormal cell proliferation in endocrine tissues, predisposing individuals to a range of endocrine and non-endocrine tumors. MEN1 is classically associated with tumors of the parathyroid glands, anterior pituitary, and enteropancreatic neuroendocrine tissues, though other endocrine manifestations can include foregut carcinoid tumors, adrenocortical tumors, and, rarely, pheochromocytoma. Non-endocrine features include meningiomas, ependymomas, lipomas, angiofibromas, collagenomas, and leiomyomas [1].

According to MEN1 clinical practice guidelines, a patient is diagnosed with MEN1 by meeting one of the following three criterion: the manifestation of at least two primary MEN1-associated endocrine tumors (i.e., parathyroid adenoma, enteropancreatic tumor, and pituitary adenoma); the occurrence of one MEN1-associated tumor in a first degree relative of a patient with a clinical diagnosis of MEN1; and the detection of a germline MEN1 mutation in an individual, who may be asymptomatic without biochemical or radiological evidence of MEN1 [2].

The estimated prevalence of MEN1 ranges from one in 20,000 to one in 40,000, with no clear gender predisposition, and more than 95% of individuals carrying a pathogenic MEN1 mutation develop clinical manifestations by 40-50 years of age [1-3]. MEN1 is typically inherited in an autosomal dominant manner, but in about 10% of cases, the mutation arises sporadically as a de novo mutation [1]. Clinical presentation is highly variable and depends on the number, size, and location of tumors. Tumors can even develop simultaneously in different tissues, affecting growth, digestion, and hormonal regulation, which makes the diagnosis particularly challenging [4]. Unfortunately, there is no genotypic-phenotypic correlation in MEN1, leading to different manifestations even among family members [5].

Early identification of MEN is essential for timely management and improved clinical outcomes. Delayed recognition of MEN1 can negatively influence patient outcomes, as tumor progression to malignant stages may occur before appropriate intervention, complicating treatment and leading to an unfavorable prognosis. MEN1 gene testing in an index case can help with early diagnosis and recognition of asymptomatic carriers well before a MEN1-associated tumor can be detected clinically. However, approximately 10-30% of MEN1 families have no mutation in the MEN1 gene, and in such cases, the diagnosis of MEN1 ​​is usually established on the basis of clinical, familial, and/or genetic criteria [6].

Our case illustrates the challenges in integrating clinical, biochemical, and radiological data to reach a conclusive diagnosis and emphasizes the importance of early recognition for improving patient outcomes.

## Case presentation

A 48-year-old Caucasian man presented to the emergency department with a four-week history of progressive abdominal pain, which was severe, constant, sharp in nature, and loose stools around four times a day with no blood or mucus. His physical examination revealed generalized abdominal tenderness and guarding, and his computed tomography (CT) abdomen showed a contained small bowel perforation with tethered loops of bowel, along with non-obstructing small left renal stones. He was admitted under the surgical team and treated conservatively with intravenous (IV) antibiotics and IV fluids. His blood tests incidentally showed hypercalcemia (adjusted calcium 2.69 mmol/L; normal range: 2.20-2.60 mmol/L), which was initially attributed to dehydration. He only had a personal history of renal calculi with painless intermittent hematuria for a decade, and his family history was unremarkable.

He was re-admitted 48 hours after discharge with recurrence of epigastric pain and a raised serum amylase level (2578 unit/L; normal range: 0-100 unit/L). CT abdomen suggested pancreatic inflammatory changes and showed adhesional proximal jejunal obstruction. He was again managed conservatively under the surgical team with analgesics, including paracetamol and oral morphine, IV fluids, and antibiotics, and slowly recovered during the course of his hospital stay. His case was discussed in the radiology meeting, which concluded that the CT finding was likely a concealed jejunal perforation with surrounding inflammatory changes. Ultrasound abdomen showed a hepatic hemangioma and a hypoechoic lesion at the head of the pancreas measuring 14 x 10 x 11 mm with no gallstones. He also developed left arm thrombophlebitis with extensive thrombus in the cephalic vein and was discharged with a course of direct oral anticoagulant (DOAC).

**Figure 1:**
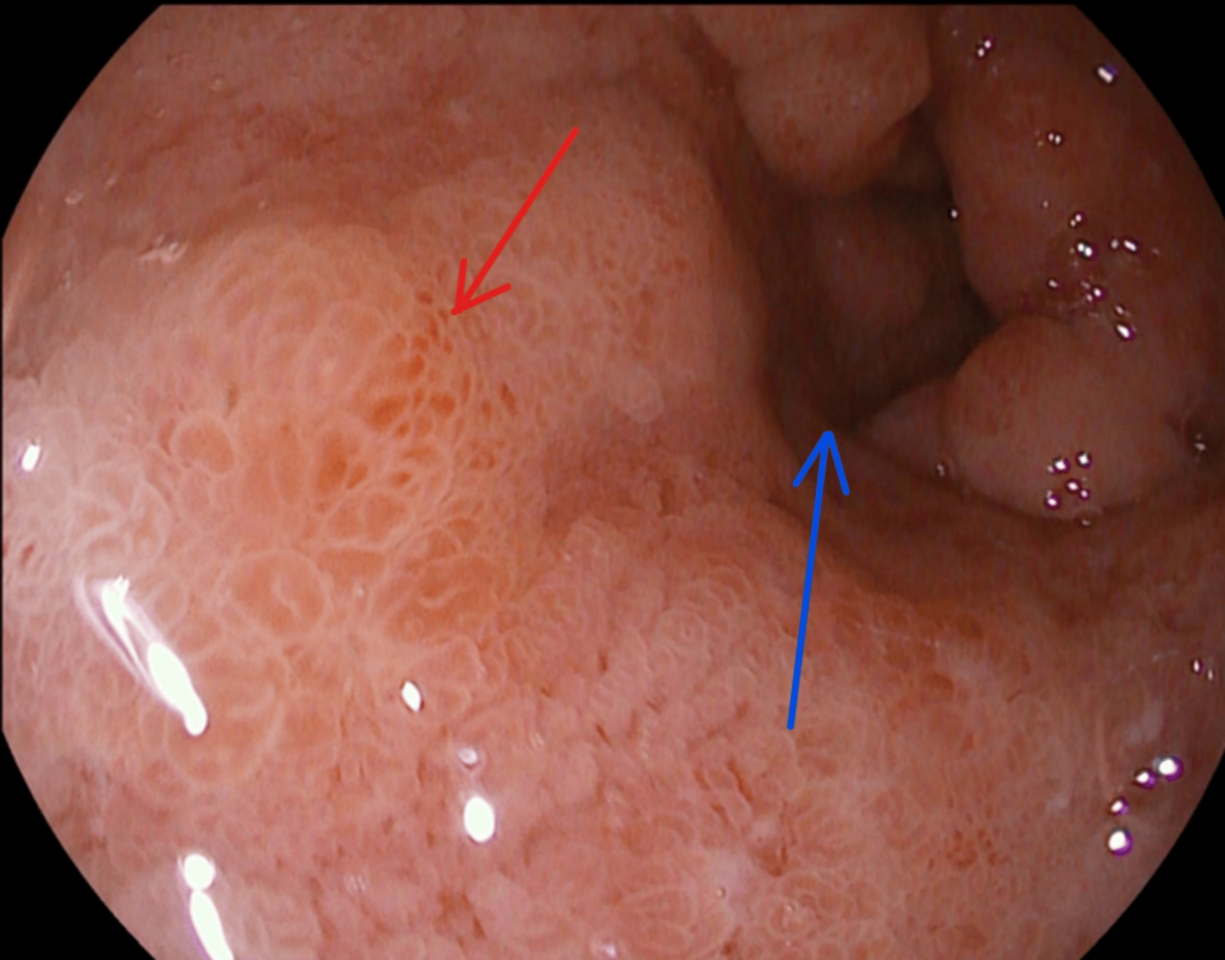
Upper GI endoscopy image 1. Duodenal submucosal edema, erosions (red arrow) and luminal narrowing (blue arrow). GI: gastrointestinal.

**Figure 2:**
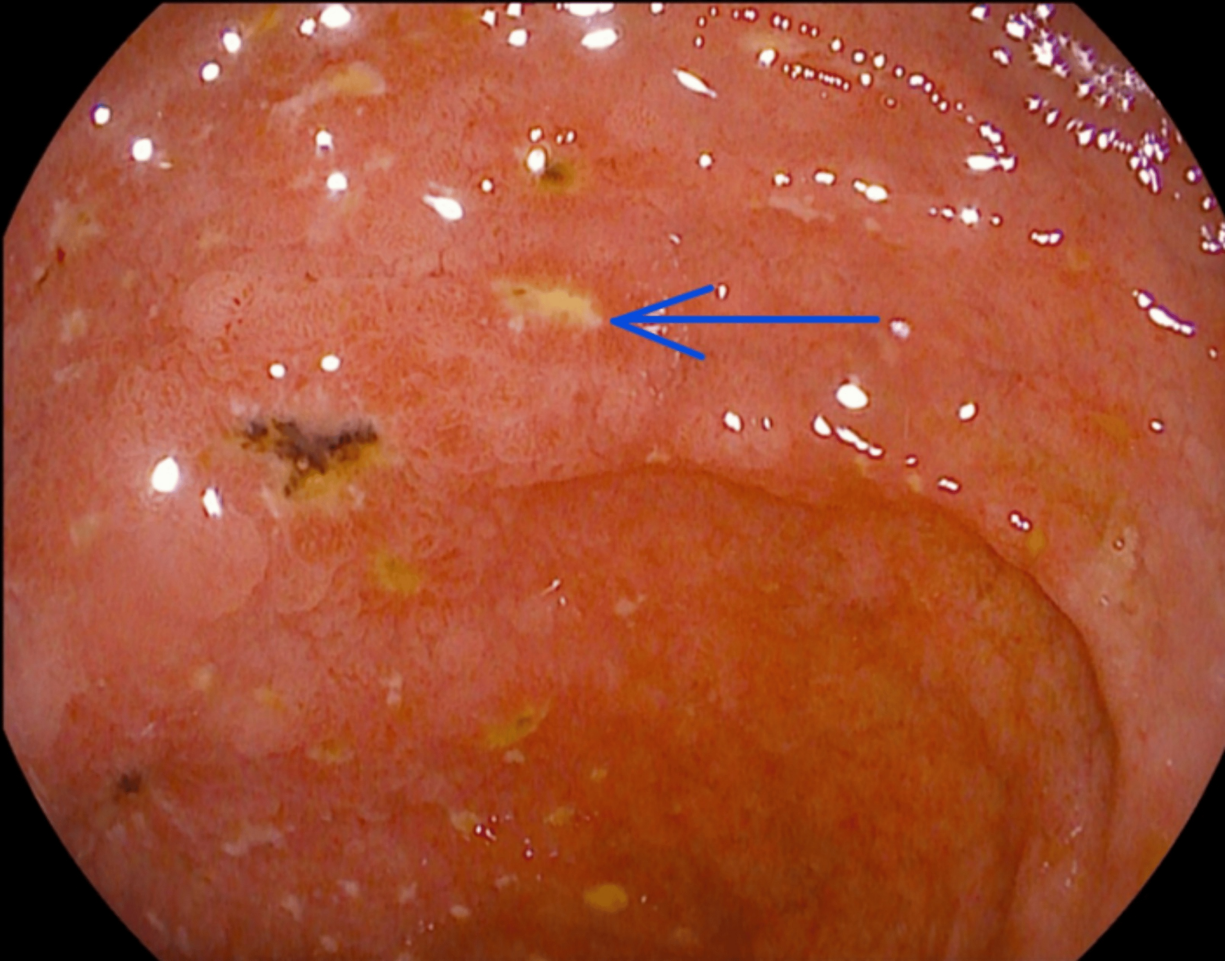
Upper GI endoscopy image 2. Duodenal ulceration (blue arrow) and erosion. GI: gastrointestinal.

**Figure 3:**
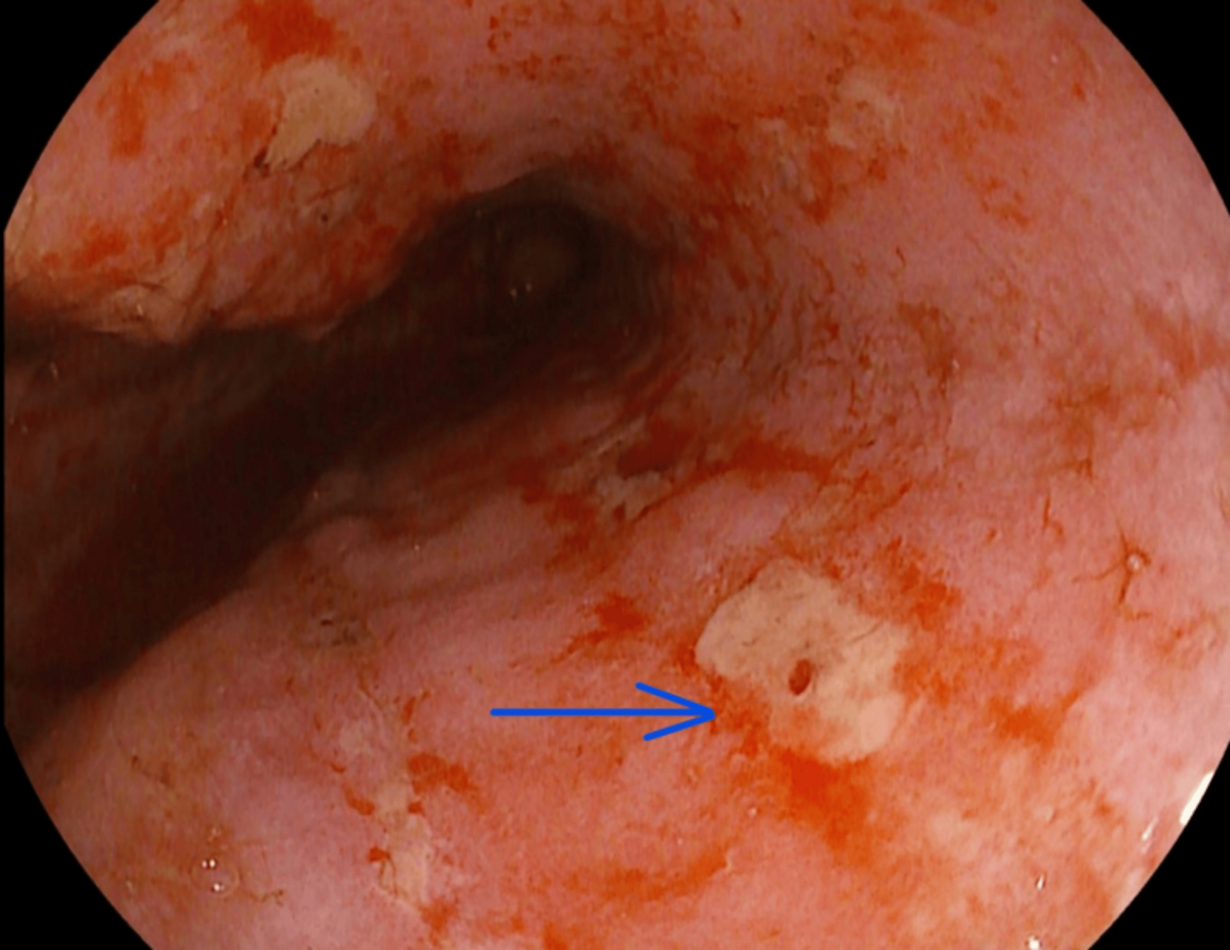
Upper GI endoscopy image 3. Severe oesophagitis with ulceration (blue arrow), erythema and edema. GI: gastrointestinal.

Within 72 hours of initiation of DOAC, he was brought in by ambulance to the hospital with hemorrhagic shock secondary to an upper gastrointestinal (GI) bleeding, having had a history of melena for two days and a hemoglobin drop from 133 g/L to 99 g/L. He received fluid resuscitation, red blood cell transfusion, IV proton pump inhibitor (PPI), and following stabilization and an unremarkable CT abdomen, underwent an upper GI endoscopy, which revealed grade Hill's grade D esophagitis and non-erosive duodenitis. He was switched to an oral high-dose PPI along with sucralfate and was cautiously restarted on DOAC in a few days. However, in the following week, he developed a second episode of GI bleed requiring blood products. Repeat gastroscopy showed a normal esophagus and an edematous duodenum with post-inflammatory changes. Considering inflammatory bowel disease (IBD), he subsequently had a colonoscopy and magnetic resonance imaging (MRI) of the small bowel, which were normal. At that point, his anticoagulation was also discontinued as a repeat ultrasound Doppler of his left arm showed resolution of the thrombus. He was trialed on a course of steroids for presumed IBD, but did not show any clinical response. Moreover, his histopathology reported Brunner's gland hyperplasia of the duodenal mucosa and patchy gastric metaplasia in the absence of atypia or any malignant features, and implied peptic duodenitis. Given his recurrent significant ulcerations, the gastroenterology team arranged for a fasting GI hormone panel to investigate further for a gastrinoma. While off PPI for two weeks prior to the testing, he was re-admitted with severe abdominal pain. Repeat CT imaging showed recurrent duodeno-jejunitis. Symptoms were managed with opioid analgesics until the fasting hormones were sent. He was discussed in the local gastroenterology-radiology multidisciplinary team (MDT) meeting and underwent a push enteroscopy for direct visualization. This showed extensive esophagitis and duodenitis, with large ulcerations in D2 and healing ulcers in D3 (Figures 1-3). Repeat histopathology report again demonstrated esophageal and duodenal inflammation, without any evidence of celiac or Crohn's disease.

Throughout his presentations, he had persistent hypercalcemia. An endocrinology consult was sought, as he had a raised parathyroid hormone (PTH) of 27.6 pmol/L (NI: 2.2-14 pmol/L) (Table 1) with low vitamin D 18.4 nmol/L (50-150 nmol/L).

**Table 1:** Blood test results. PTH: parathyroid hormone; TSH: thyroid-stimulating hormone.

Parameter	Value	Normal range
Gastrin (pmol/L)	202	<40
Adjusted calcium (mmol/L)	2.69	2.20-2.60
Inorganic phosphate (mmol/L)	0.52	0.80-1.50
PTH (pmol/L)	27.6	2.2-14
TSH (milliunit/L)	1.42	0.30-4.20
T4 (pmol/L)	14.4	9-23

**Figure 4:**
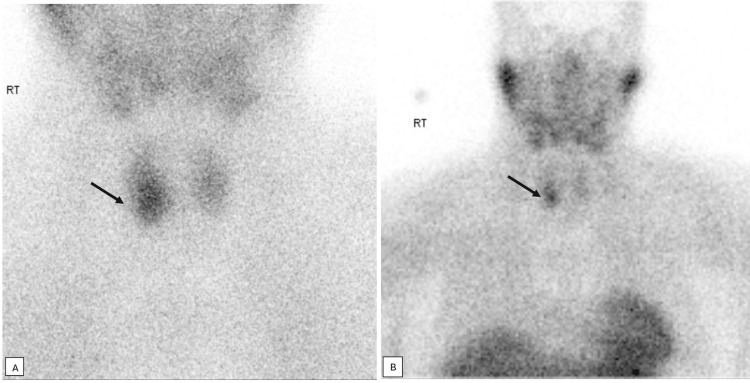
Parathyroid SPECT. 11 mm soft tissue nodule (black arrows) abutting the right cervical paravertebral muscles posteriorly and the trachea medially at the level of right mid/lower pole thyroid, with increased tracer uptake. SPECT: single-photon emission computed tomography.

A subsequent parathyroid single-photon emission computed tomography (SPECT) showed an 11 mm right lower parathyroid adenoma (Figure 4). He was subsequently commenced on cinacalcet, which is a calcimimetic that heightens the parathyroid gland's sensitivity to circulating calcium by allosterically activating the calcium-sensing receptor, leading to reduced parathyroid hormone release.

**Figure 5:**
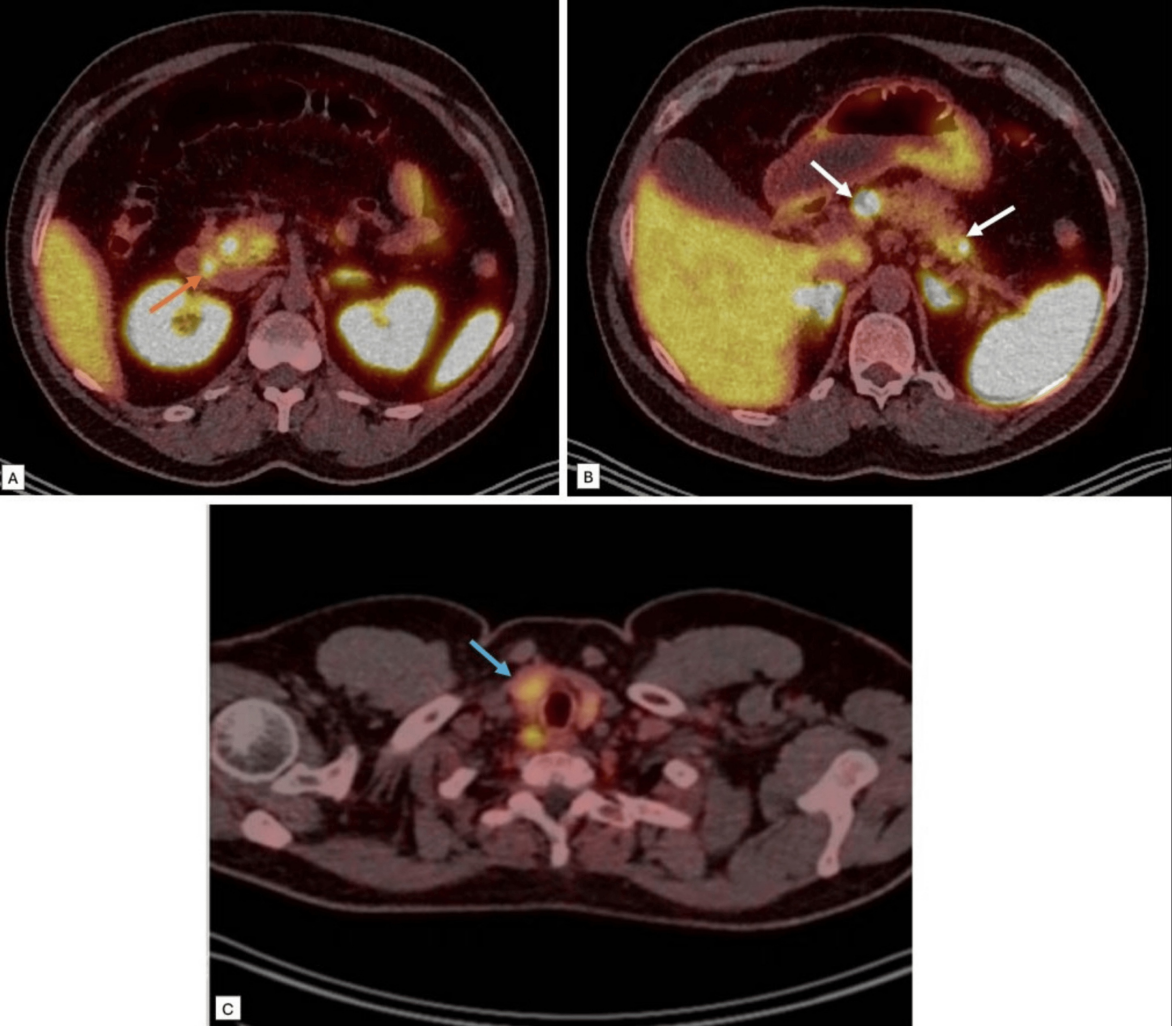
Ga68 DOTA-TATE whole body PET-CT. Multiple foci of intense tracer uptake in the pancreas (white arrows in image (B)) and a focus of uptake in the duodenum (orange arrow in image (A)), and the right inferior parathyroid adenoma (blue arrow in image (C)). PET: positron emission tomography.

The fasting GI hormone panel showed significantly raised gastrin levels of 202 pmol/L (NI <40 pmol/L) (Table 1); however, the remainder of the gut hormone profile was normal. He was subsequently started on a high-dose PPI and referred to the regional neuroendocrine MDT, and following their recommendation, had a Ga68 DOTA-TATE whole body positron emission tomography (PET) CT done, which revealed multiple metastatic receptor-expressing tumors within the pancreas, with a focus of uptake in the duodenum and in the right inferior parathyroid adenoma (Figure 5).

Notably, our patient did not exhibit any signs or symptoms of hypopituitarism or hyperpituitarism, and his pituitary profile was normal. Genetic testing confirmed suspicions of MEN1 syndrome and showed MEN1 heterozygous Cys241Tyr.

He was then planned for a subtotal parathyroidectomy, following which the hepatobiliary surgical team planned to undertake a total pancreatectomy with duodenectomy (Whipple's procedure). His children have been referred for genetic testing. He will be followed up by the endocrinology and the hepatobiliary team.

## Discussion

This case highlights the diagnostic complexity of MEN1, particularly when initial manifestations mimic more common GI conditions. Our patient presented with recurrent GI ulceration and bleeding, which was initially attributed to IBD or peptic ulcer disease. Only after repeated admissions, elevated fasting gastrin levels, and biochemical evidence of hypercalcemia with raised parathyroid hormone (PTH) was MEN1 suspected and subsequently confirmed on genetic testing.

In MEN1, pancreatic neuroendocrine tumors (pNETs) are the second most common neoplasms after parathyroid adenomas, affecting 30-80% of patients, and are a leading cause of MEN1-related mortality [2]. Functional imaging plays a critical role in staging these tumors and characterizing the primary lesion, particularly when pNETs present with metastases. The European Neuroendocrine Tumor Society (ENETS) guidelines recommend the use of gallium-PET-DOTA-TATE PET-CT tumor staging and assessment of somatostatin receptor expression in pNETs ≥1 cm or in lesions demonstrating growth [7]. In this case, DOTA-TATE PET-CT results revealed multifocal somatostatin receptor-positive lesions within the pancreas and duodenum, consistent with multifocal gastrinoma. Integration of biochemical, genetic, and molecular imaging findings confirmed the diagnosis and guided surgical planning.

The patient's hyperparathyroidism and associated clinical features represent the most common and earliest manifestation of MEN1 [8]. Elevated calcium levels potentiate gastrin release, thereby worsening acid hypersecretion and ulcer formation. This synergistic relationship between the parathyroid and involvement of the pancreas in MEN1 likely contributed to his severe peptic disease and recurrent bleeding. Accordingly, a subtotal parathyroidectomy was planned as definitive management of his hyperparathyroidism.

Interestingly, this patient had no evidence of pituitary disease, consistent with the variable penetrance observed in MEN1. Pituitary adenomas occur in approximately 15-50% of patients, but the absence of biochemical or radiological abnormalities does not preclude future development, thereby necessitating regular screening [9,10].

The episode of thrombophlebitis can be explained by the high thrombotic burden in neuroendocrine tumors, particularly those that are functional and of pancreatic origin [11]. Genetic analysis confirmed a heterozygous pathogenic variant in MEN1 (c.722G>A; p.Cys241Tyr). Therefore, genetic counseling was arranged for the individual's children, aligning with the current consensus that all first-degree relatives of affected individuals should screening for MEN1 [10]. In our patient, the absence of a family history of MEN1 may reflect a de novo mutation or the presence of asymptomatic or minimally symptomatic parents, as MEN1 is known to have variable penetrance. Pancreatic resection is indicated in patients with functional pNETs due to the risk of malignant transformation and metastatic potential, as well as to reduce symptoms caused by hormone hypersecretion or local invasion [12]. High-dose PPI should be given in a gastrinoma and would have to continue for several months following successful surgery [12]. A multidisciplinary approach is crucial with early involvement of a hepatobiliary surgeon, gastroenterologists, radiologists, and endocrinologists.

In summary, this case emphasizes the importance of considering MEN1 in patients with refractory or recurrent GI ulceration, particularly in the presence of hypercalcemia or pancreatic lesions. Early suspicion, recognition, and a multidisciplinary approach to management are pivotal in preventing end-organ damage and improving the prognosis of MEN1.

## Conclusions

This case presents the clear diagnostic challenges posed by MEN1 when classic family history is absent. Recurrent GI ulcerations with bleeding, along with hypercalcemia, represented early but nonspecific manifestations that initially mimicked more common GI diseases, resulting in diagnostic delay. Early suspicion, targeted biochemical, genetic testing and functional imaging were essential in reaching the diagnosis and planning for definitive management. Early recognition of MEN1 is crucial, as timely management can reduce the risk of malignant transformation and metastasis and also enables early screening of family members. This case highlights the need for clinicians to consider MEN1 even when clinical features are atypical, family history is not evident or overlap with more common GI conditions.

## References

[1] Kamilaris CD, Stratakis CA (2019). Multiple endocrine neoplasia type 1 (MEN1): an update and the significance of early genetic and clinical diagnosis. Front Endocrinol (Lausanne).

[2] Thakker RV, Newey PJ, Walls GV (2012). Clinical practice guidelines for multiple endocrine neoplasia type 1 (MEN1). J Clin Endocrinol Metab.

[3] Singh R, Goel SA, Singh JS, John DR, Suthar PP (2025). Multiple endocrine neoplasia type 1 (MEN1) syndrome clinical presentation and the role of newer functional imaging in the diagnosis and management: a case report. Cureus.

[4] Giusti F, Marini F, Brandi ML (1993). Multiple endocrine neoplasia type 1. GeneReviews®.

[5] Singh G, Mulji NJ, Jialal I (2023). Multiple endocrine neoplasia type 1. StatPearls (Internet).

[6] de Laat JM, van der Luijt RB, Pieterman CR (2016). MEN1 redefined, a clinical comparison of mutation-positive and mutation-negative patients. BMC Med.

[7] Carli A, Boffa E, Bonatti M, Chincarini M, Davì MV, Zamboni GA (2025). Multimodal imaging approach to MEN-1 syndrome-associated tumors. Diagnostics (Basel).

[8] Brandi ML, Gagel RF, Angeli A (2001). Guidelines for diagnosis and therapy of MEN type 1 and type 2. J Clin Endocrinol Metab.

[9] Vergès B, Boureille F, Goudet P (2002). Pituitary disease in MEN type 1 (MEN1): data from the France-Belgium MEN1 multicenter study. J Clin Endocrinol Metab.

[10] Brandi ML, Pieterman CRC, English KA (2025). Multiple endocrine neoplasia type 1 (MEN1): recommendations and guidelines for best practice. Lancet Diabetes Endocrinol.

[11] Massironi S, Gervaso L, Fanizzi F, Preatoni P, Dell'Anna G, Fazio N, Danese S (2025). Venous thromboembolism in patients with neuroendocrine neoplasms: a systematic review of incidence, types, and clinical outcomes. Cancers (Basel).

[12] de Ponthaud C, Menegaux F, Gaujoux S (2021). Updated principles of surgical management of pancreatic neuroendocrine tumours (pNETs): what every surgeon needs to know. Cancers (Basel).

